# Differentially Expressed Extracellular Vesicle-Contained microRNAs before and after Transurethral Resection of Bladder Tumors

**DOI:** 10.3390/cimb43010024

**Published:** 2021-06-04

**Authors:** Olaf Strømme, Kathleen A. Heck, Gaute Brede, Håvard T. Lindholm, Marit Otterlei, Carl-Jørgen Arum

**Affiliations:** 1 Department of Clinical and Molecular Medicine, Norwegian University of Science and Technology, 7491 Trondheim, Norway; kathleen.heck@ntnu.no (K.A.H.); gaute.brede@ntnu.no (G.B.); marit.otterlei@ntnu.no (M.O.); carl-jorgen.arum@ntnu.no (C.-J.A.); 2 CEMIR—Centre of Molecular Inflammation Research, Department of Clinical and Molecular Medicine, Norwegian University of Science and Technology, 7491 Trondheim, Norway; havard.t.lindholm@ntnu.no; 3 Department of Urology, St. Olav’s University Hospital, 7030 Trondheim, Norway

**Keywords:** biomarkers, bladder cancer, exosome, extracellular vesicles, liquid biopsy, miRNA, non-muscle invasive bladder cancer, pre/postsurgery, transurethral resection of bladder tumor

## Abstract

Bladder cancer (BC) is currently diagnosed and monitored by cystoscopy, a costly and invasive procedure. Potential biomarkers in urine, blood, and, more recently, extracellular vesicles (EVs), have been explored as non-invasive alternatives for diagnosis and surveillance of BC. EVs are nanovesicles secreted by most cell types containing diverse molecular cargo, including different types of small RNAs, such as microRNA (miRNA). In this study, we performed next-generation sequencing of EV-contained miRNA isolated from urine and serum of 41 patients with non-muscle invasive BC (27 stage Ta, 14 stage T1) and 15 non-cancer patients (NCP) with benign cystoscopy findings. MiRNA sequencing was also performed on serum supernatant samples for T1 patients. To identify potential BC-specific biomarkers, expression levels of miRNA in presurgery samples were compared to those at postsurgery check-ups, and to NCPs. Results showed that two miRNAs, urinary EV-contained miR-451a and miR-486-5p, were significantly upregulated in presurgery samples from T1 patients compared to postsurgery check-up samples. This was confirmed in a replica EV/RNA isolation and sequencing run of 10 T1 patients from the primary run; however, analyses revealed no differential expression of miRNAs in serum EVs, serum supernatant, or when comparing BC patients to NCPs. This is the first study to investigate EV-containing miRNA sequencing in pre- and postsurgery BC patient samples and our findings suggest that urinary EV-contained miR-451a and miR-486-5p may be potential biomarkers for recurrence-free survival of BC patients with stage T1 disease.

## 1. Introduction

Bladder cancer (BC) is the second most common urological cancer with more than 500,000 new cases annually worldwide [1]. Due to frequent recurrences, monitoring, and repeated surgical interventions, BC has been estimated to be among the most expensive cancers to treat over the lifetime of a patient. Cystoscopy, the primary tool for diagnosis and surveillance of BC, is a costly invasive procedure that causes discomfort and an elevated risk of urinary tract infections [2,3]. Urine cytology is a non-invasive diagnostic alternative routinely used in the clinic, but low sensitivity, particularly for low-grade tumors, restricts its utility [4,5,6]. Thus, there is a need for non-invasive biomarkers that can limit the need for cystoscopy in the diagnosis and surveillance of BC.

Extracellular vesicles (EVs) are a collective term for membrane-bound nanosized vesicles. Exosomes are small EVs, typically 40–100 nm in diameter, that originate from multivesicular bodies of the endocytic pathway [7,8]. Microvesicles (MVs) are more heterogeneous in size, (100–1000 nm in diameter), and are secreted through a budding process of the plasma membrane [9,10]. Both types of EVs consist of a lipid bilayer enveloping diverse types of intravesicular cargo such as peptides, metabolites, and RNA, including small RNAs (sRNA) such as microRNA miRNA [11,12,13]. Upon secretion to the extracellular space, EVs may be taken up by neighboring cells or diffuse into the systemic circulation, potentially reaching tissues distant from their cells of origin [14]. Through the incorporation of the EV-cargo, the phenotype of the recipient cell may be altered, as can be demonstrated through the transfer of malignant properties from one cell to another within a tumor population [15]. As the intravesicular molecular cargo is protected from degradation, EVs isolated from bodily fluids such as urine and serum are considered promising reservoirs of biomarkers for various cancers, including BC [16,17,18].

EVs contain significant amounts of miRNAs, ~22-nucleotide non-coding sRNAs, that play a pivotal role in gene regulation by silencing post-transcriptional gene expression. By regulating a large repertoire of both proto-oncogenes and tumor-suppressor genes, miRNAs play an important role in the oncogenesis of many types of cancer, including BC [19,20]. It has been shown that miRNAs contained within EVs (EVmiRNAs) can be shuttled between cells and that EVmiRNAs may regulate gene expression upon uptake by recipient cells [21]. EVmiRNAs are believed to play an important role in carcinogenesis [22,23] and may have promise as diagnostic and prognostic cancer biomarkers [24,25]. A few studies have explored EVmiRNAs as potential biomarkers in BC. Baumgart et.al investigated the miRNA profiles of EVs derived from cultured NMIBC and MIBC cells and found that a distinct EVmiRNA profile could distinguish the two BC subtypes [26]. Andreu et al. identified urinary EV-contained miR-375 and miR-146a as biomarkers for high-grade and low-grade BC respectively [27], whereas Matsuzaki et al. identified miR-21-5p as a diagnostic BC biomarker in patients with negative urine cytology [28]. As of yet, no study has investigated the differential expressions of EVmiRNAs before and after transurethral resection of bladder tumors (TURB).

In this study, next-generation sequencing (NGS) was performed for EV-contained sRNAs isolated from urine and serum of 41 patients with non-muscle invasive BC (NMIBC) and 15 non-cancer patients (NCPs) with benign cystoscopy findings. To identify potential BC-specific biomarkers, expression levels of miRNAs in presurgery samples were compared to those at postsurgery check-ups, and to NCPs. Among BC patients with stage T1 disease, two urinary EVmiRNAs, miR-451a and miR-486-5p, were significantly upregulated presurgery compared to at postsurgery check-up where no recurrence was detected. MiR-451a and miR-486-5p may thus represent potential biomarkers for recurrence-free survival of BC patients with stage T1 disease.

## 2. Results

### 2.1. Patient Characteristics

**Figure 1:**
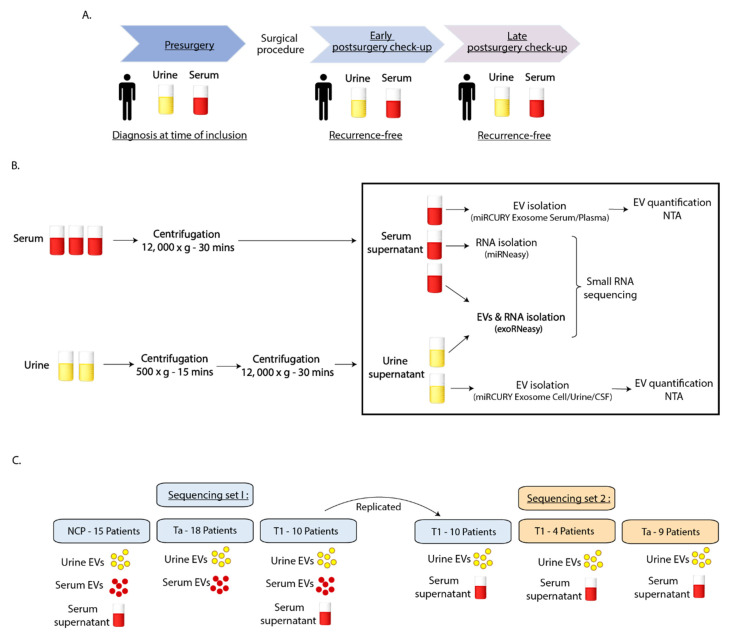
Study outline and sample workflow. (**A**) BC patients donated urine and blood samples prior to BC surgery and at an early and a late postsurgery check-up. Not all patients had a late postsurgery sample included. (**B**) Workflow from sample collection to EV quantification and small RNA sequencing. (**C**) The study consisted of two sequencing sets. Sequencing set 1 included urine EVs, serum EVs, and serum supernatant, while sequencing set 2 was limited to urine EVs and serum supernatant. Samples from ten T1 patients were run as replicas in sequencing set 2.

Serum and urine samples were collected from 41 BC patients and 15 NCPs fulfilling the study inclusion criteria. An overview of patient characteristics is provided in Table 1 for BC patients and Table S1 for the NCPs. The primary diagnosis and diagnosis at the time of inclusion (TOI) in the study are shown respectively. In the BC group, there were 32 males and nine females, and the average age at inclusion was 72.3 years. In the NCP group, there were seven males and eight females, and the average age at inclusion was 63.5 years. In the BC group, 27 patients were stage Ta, and 14 were stage T1 at the time of inclusion into the study. Six of the BC patients underwent intravesical bacille Calmette-Guérin (BCG) treatment (Table S2). Three of the T1 patients underwent cystectomy prior to check-up sampling following TURB. A description of each BC patient-derived sample is shown in Table S3, including the diagnosis at the time of inclusion, clinical procedure, as well as the interval between samples. Samples were collected prior to TURB, and at one or two postsurgery check-ups where no recurrence was detected (Figure 1A). Samples were prepared in order to carry out sRNA sequencing (Figure 1B,C).

### 2.2. Biosource Separation Based on Principal Component Analysis

Principal component analysis (PCA) of miRNAs identified in sequencing set 1 and sequencing set 2 from urinary EVs, serum EVs, and serum supernatant showed clear clustering of the three distinct biosources (Figure S1). Samples were more uniformly distributed for serum than for the EV biosources, with urinary EVs displaying the most variation. Taken together, the clustering of biosources indicated that laboratory steps were successfully carried out, enabling analyses exploring biological differences between sample groups.

### 2.3. EV Characterization

Size distribution and particle concentration for EVs isolated from urine and serum were measured by Nanoparticle Tracking Analysis (NTA) and are displayed for BC patients (Table S4) and NCPs (Table S1). The sample concentration measured in particles/mL and average mode sample size (nm) was presented for each individual sample. An average mode particle size of 78 nm (urine EVs) and 45 nm (serum EVs) in BC patients was observed, consistent with an EV population mostly represented by small EVs [29,30]. Representative NTA graphs for urine and serum EVs from BC patients and NCPs are found in Figures S2 and S3.

### 2.4. Differential Expression Analysis Reveals Potential MiRNA Biomarkers for T1 BC Patients

**Figure 2:**
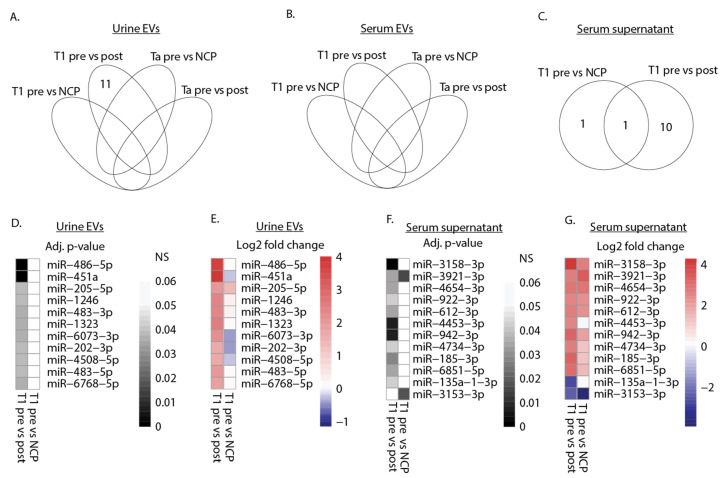
Differential expression of miRNAs in urine EVs and serum supernatant. (**A**–**C**) Venn diagram depicting the number of DEmiRNAs in presurgery (pre) samples compared to postsurgery (post) samples and NCPs in urine EVs, serum EVs, and serum supernatant. (**D**,**E**) Heat map displaying adjusted *p*-values and log2 fold change values for the eleven DEmiRNAs detected in (**A**) urine EVs. miRNA expression levels were compared between T1 presurgery samples and postsurgery samples or NCPs. (**F**,**G**) Heat map displaying adjusted *p*-values and log2 fold change values for the twelve DEmiRNAs detected in (**C**) serum supernatant. miRNA expression levels were compared between T1 presurgery samples and postsurgery samples or NCPs. An adjusted *p*-value threshold of 0.05 was set to determine DEmiRNAs for all analyses and non-significance (NS) is denoted in the scale.

Differential expression analysis was performed on the complete set of patients sequenced in sequencing set 1 and in sequencing set 2. MiRNAs were defined as differentially expressed (DEmiRNAs) between sample groups if an adjusted *p*-value of <0.05 was obtained. In total, eleven DEmiRNAs were found in urinary EVs, twelve DEmiRNAs were identified in the serum supernatant, while analysis of serum EVs did not reveal DEmiRNAs (Figure 2A–C). Most of the DEmiRNA in both urine EVs and serum supernatant were found when comparing expression profiles before and after BC surgery. Only in serum supernatant were two DEmiRNAs identified when comparing BC patients to NCPs. DEmiRNAs were exclusively identified in T1 patients, and not in Ta patients, indicating that the expression of dysregulated miRNAs may be positively correlated to more advanced tumor stages. The adjusted *p*-value and log2 fold change for DEmiRNAs were plotted in the heat maps (Figure 2D–G) and numerical values shown (Table S4). The number of the fourteen T1 patients where the highest amount of miRNA was found in the presurgery sample for each individual DEmiRNA in urine EVs and serum supernatants are listed in Table 2. All DEmiRNAs in urine EVs were upregulated in T1 presurgery samples compared to postsurgery samples, and this trend was observed in serum supernatant as well.

### 2.5. Replica Sequencing Confirms Differential Expression of miR-451a and miR-486-5p in Urinary EVs

**Figure 3:**
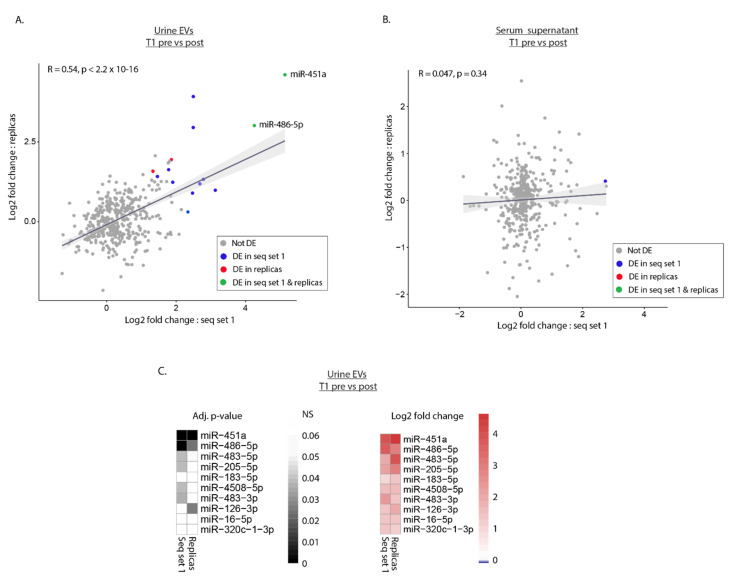
Reproducibility of sequencing data for T1 patients. (**A**,**B**) Dot plot showing by log2 fold change for miRNAs in urine EVs (**A**) and serum supernatant (**B**) in T1 presurgery (pre) versus postsurgery (post) samples. Samples from ten T1 patients from sequencing (seq) set 1 are compared to replicas from the same patients. Each dot represents an individual miRNA detected in both runs. Dot colors indicate whether miRNAs are DE in seq set 1, replicas, or in both. (**C**) Heat maps showing adjusted *p*-values and log2 fold change values for the top ten DEmiRNAs in urine EVs in T1 pre- versus postsurgery samples from seq set 1. An adjusted *p*-value threshold of 0.05 was set to determine DEmiRNAs for all analyses and non-significance (NS) is denoted in the scale.

To assess the reproducibility of our results, EV/RNA isolation, and subsequent sRNA sequencing were performed on new urine and serum supernatant aliquots for the ten T1 patients that were sequenced in sequencing set 1 as part of sequencing set 2 (Figure 1C). Log2 fold change values between presurgery T1 samples and recurrence-free postsurgery samples were calculated for annotated miRNAs in both sequencing runs. For urinary EVs, a significant correlation was observed between sequencing set 1 and replica samples in sequencing set 2, as demonstrated by an R-squared value of 0.54 (Figure 3A). For serum supernatant, none of the DEmiRNAs identified in sequencing set 1 were confirmed in the replica run, consistent with a poor R-squared value of 0.047 (Figure 3B). Two miRNAs, miR-451a and miR-486-5p, were differentially expressed in both sequencing runs, displaying similarly adjusted *p*-values and log2 fold change (Figure 3C and Table S5). As only ten patients are included in the replication run (Figure 3C) and 14 are included in the complete analysis (Figure 2D,E) there are differences in the miRNA hits with a *p*-value close to the threshold obtained in the analysis of the complete data set (Figure 2D,E) compared to the analysis of only patients sequenced in both sequencing sets (Figure 3C).

### 2.6. miR-451a and miR-486-5p Are Highly Expressed in Presurgery Urinary EVs among T1 Patients

**Figure 4:**
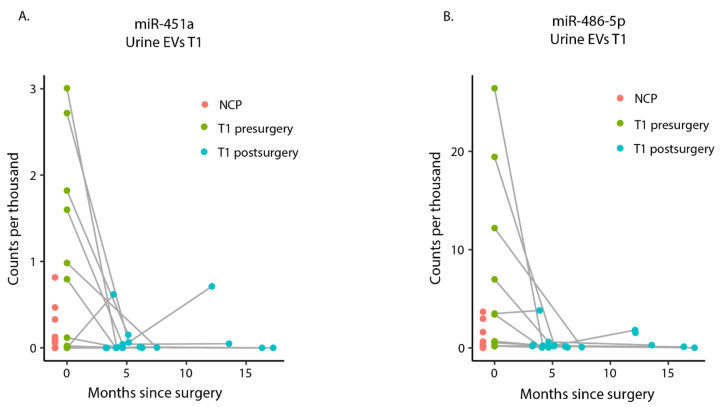
Expression levels of miR-451a and miR-486-5p in presurgery and postsurgery samples. (**A**) Line plot showing miR-451a expression in counts per thousands (CPT). Each line follows one T1 patient from presurgery cancer sample (green) to early and late postsurgery samples (blue). (**B**) Same observation as highlighted in (**A**) for miR-486-5p.

Both DEmiRNAs, miR-451a, and miR-486-5p, were upregulated in presurgery samples compared to postsurgery. The plotting of the raw count per thousand (CPT) values for miR-451a and miR-486-5p revealed that for a subset of T1 patients both miRNAs were upregulated in urinary EVs in presurgery samples compared to the postsurgery samples in 7 and 5 out of 14, respectively (Figure 4). We could not find any patient variables, such as sex, smoking status, or BCG treatment that could predict which patients would belong to this subset of T1 patients; however, larger sample sizes may be needed to identify characteristics of this subgroup. Interestingly, even though miR-451a (Figure 4A) and miR-486-5p (Figure 4B) were not statistically significantly upregulated in presurgery samples compared to NCP samples, the NCP samples still show a lower overall expression.

### 2.7. miR-451a and miR-486-5p Are Involved in Transcription Regulation, Cell Differentiation, and Apoptotic Signaling

**Figure 5:**
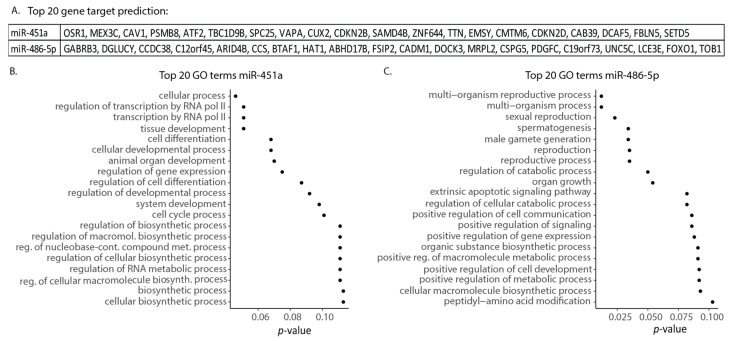
Gene target predictions and Gene Ontology term analysis for miR-451a and miR-486-5p. (**A**) The top 20 gene target predictions according to the R package miRNAtap for miR-451a and miR-486-5p are shown. (**B**,**C**) Top 20 Gene Ontology (GO) term categories for miR-451a (**B**) and miR-486-5p (**C**) using the R package ‘topGO’ based on predicted targets.

To explore the biological relevance of miR-486-5p and miR-451a, we performed Gene Ontology (GO) term analysis and Gene Target predictions (Figure 5). The R package, miRNAtap, was applied in order to obtain predicted gene targets for the two miRNAs, and the top 20 gene target predictions are shown (Figure 5A). MiR-451a gene targets involve (i) *cellular signaling* such as caveolin 1 (CAV1, Ras-ERK signaling), calcium-binding protein 39 (CAB39, binding and activation) VAMP-Associated Protein A (VAPA, membrane trafficking), and Sterile alpha motif domain containing 4B (SAMD4B, transcriptional repressor) and (ii) *cell cycle regulation and transcription* such as activating transcription factor 2 (ATF2, transcription factor (bind CRE) and histone acetyltransferase), kinetochore protein Spc25 (SPC25, chromosome segregation), cyclin-dependent kinase 4 inhibitor B (CDKN2B) and D (CDKN2D) (cell cycle regulation). For miR-486-5p, the top 20 gene targets were found to be involved in (i) *cellular transcription* including AT-rich interactive domain-containing protein 4B (ARID4B, part of transcriptional co-repressor complex), TAT-binding protein-associated factor 172 (BTAF1, transcription initiation), and forkhead box protein 01 (FOXO1, transcription factor) as well as (ii) *replication* e.g., Histone acetyltransferase 1 (HAT1, chromosome assembly) and (iii) *cell adhesion*, e.g., cell adhesion molecule 1 (CADM1).

The miRNA target predictions were applied to predict GO terms using the R package topGO (Figure 5B,C). For miR-451a, the GO terms with the lowest *p*-value are shown for transcription regulation, tissue development, and cellular processes. GO terms linked to the cell cycle process and regulation of cell differentiation also appeared. For miR-486-5p, the GO terms with the lowest *p*-value were linked to sexual reproduction. However, GO terms associated with miR-486-5p also included regulation of cellular signaling, gene expression, and apoptotic signaling.

## 3. Discussion

Previous studies investigating EVmiRNAs as biomarkers have predominantly compared the expression of miRNAs in urine EVs of BC patients to those of healthy controls [29,30]. This is to the best of our knowledge the first study that compares expression profiles of EVmiRNAs in urine and serum before and after TURB. By using this approach, we identified two EVmiRNAs, miR-451a and miR-486-5p, which were significantly upregulated in presurgery urine samples in T1 patients compared to postsurgery samples. Although a larger sample size would be advantageous to solidify our findings, this study suggests that miR-451a and miR-486-5p may be potential biomarkers for recurrence-free survival of BC patients with stage T1 disease.

The upregulation of miR-451a and miR-486-5p in presurgery samples was confirmed in a replica EV/RNA isolation and sequencing run, showing consistent log2Fold change and adjusted *p*-values (Table S5). MiR-451a has previously been described as a tumor suppressor in several cancers [31,32,33], including BC [34,35]. Consistent with this observation, miR-451a is found to be downregulated in BC tissue [36,37], while it has been shown to be upregulated in the urine of muscle-invasive BC (MIBC) patients [38]. It might seem surprising that the tumor suppressor, miR-451a, is upregulated presurgery compared to postsurgery in our study. However, it has been proposed that miR-451a is selectively enriched in small EVs compared to the cells of origin [39,40], potentially as a way for the tumor cell to get rid of this tumor-suppressive miRNA. Selective enrichment of miR-451a has been reported in EVs derived from oral cancer cells [39] and non-small cell lung cancer (NSCLC) cells [40]. Interestingly, exosomal miR-451a has been found to be upregulated in the urine of prostate cancer patients [41] and in the serum of NSCLC and pancreatic cancer patients as compared to healthy controls [42,43].

MiR-486-5p has also been reported to possess tumor-suppressor properties in malignancies such as lung cancer [44] and breast cancer [45], while both oncogenic and tumor-suppressor functions have been reported for NSCLC [46,47] and prostate cancer [48,49]. To date, we have found only three publications that have studied miR-486-5p in BC. While two studies reported that overexpression of mir-486-5p increased BC cell sensitivity to cisplatin [50,51], a comprehensive NGS study of urinary miRNAs in BC patients and healthy controls found that miR-486-5p was significantly upregulated in the BC group and miR-486-5p was included in a proposed three-miRNA diagnostic panel [38]. MiR-486-5p expression was found to be positively correlated to the BC stage where the highest expression was noted in MIBC patients. It remains to be seen whether miR-486-5p upregulation in urinary EVs, presurgery compared to postsurgery, may be more pronounced in advanced stages of BC. Gene ontology term analysis showed that miR-451a and miR-486-5p are involved in cellular functions important in cancer development such as cell signaling, cell differentiation, and cell cycle regulation, highlighting their potential relevance in carcinogenesis.

We detected CAB39 and CDKN2D in our target prediction for miR-451a. These two proteins are involved in cellular energy regulation (CAB39/AMPK pathway) and cell cycle progression (CDKN2D). Studies in Glioblastoma cellular proliferation and migration for both of these proteins identified the potential of therapeutically targeting miR-451 in order to inhibit cell growth and slow down cell migration through the blocking of the CAB39/AMPK pathway [52,53]. We also identified targets for miR486-5p including HAT1 and FOXO which are implicated in cell cycle and cellular metabolism and become disrupted in disease. A study by Zhang et al. found that miR-486-5p can increase the inflammatory response in chronic obstructive pulmonary disease patients by targeting HAT1 [54]. MiR-486-5p has been found to suppress key signaling proteins including FOXO1, which can contribute to prostate cancer progression [49]. Additional studies to better understand the role of miR-451a and miR-486-5p in BC are needed.

We did not find any difference in miR-451a and miR-486-5p expression between BC patients and NCPs. Consequently, the study does not suggest a role for EVmiRNAs in primary BC diagnosis. However, the significant decrease in expression of these two miRNAs in postsurgery samples suggest they may hold potential as biomarkers of recurrence-free survival.

In contrast to Sabo et al. who identified several DEmiRNAs in plasma EVs from BC patients compared to healthy controls [55], we did not identify DEmiRNAs in serum EVs, neither when presurgery samples were compared to postsurgery samples nor to NCPs. Serum EVs are highly diverse, originating from heterogeneous types of cells from multiple organ systems. Thus, a potential explanation for the lack of identified DEmiRNAs in serum EVs could be that BC-derived EVs constitute an insufficient proportion of the total serum EV population for BC-specific signatures to be revealed. In contrast, BC-derived EVs are expected to account for a larger proportion of the total urine EV population, as urine EVs are mainly secreted from cells of the urinary tract.

Surprisingly for urinary EVs, no DEmiRNAs were identified when comparing presurgery samples to NCPs, and when comparing EVmiRNA expression before after BC surgery, the differential expression was only found in the T1 group. This is somewhat contrary to previous studies that have identified differentially expressed urinary EVmiRNAs between BC patients and healthy controls [27,28,56]. Notably, our control group differs from the aforementioned studies, as it consists of NCPs who all presented with benign bladder pathology. Moreover, different EV/RNA isolation methods have been shown to produce divergent results [57], cautioning against direct comparisons of our results to those using other EV isolation methods.

While we argue this study presents a novel approach in the search for BC biomarkers, we also recognize some important limitations. Firstly, despite including a total of 41 BC patients, the T1 group, where we found the most interesting results, consisted of only 14 patients, potentially limiting the number of DEmiRNAs identified. Thus, future studies should aim to include a larger sample size of T1 patients. Secondly, for the purpose of identifying primary diagnostic BC biomarkers, an age- and sex-matched healthy control group should be included. Furthermore, this study did not include spike-in miRNA normalization to control for variations in yield from the miRNA extraction. Spike-in miRNA added to biofluids would be lost when extracting exosomes and the total miRNA isolated was too low for accurate quantification and consequent calibration of the amounts of spike-in miRNA to add. Lastly, we have not attempted to identify the source of EV-contained miR-451a and miR-486-5p in urine. Both miRNAs are known to be secreted by BC cells [51,58] but are also expressed by other cells, including erythrocytes [59].

This study also has several strengths. It is to the best of our knowledge the first study to compare EV-contained miRNAs profiles before and after BC surgery. By including two control groups, both recurrence-free controls from the same patient, and benign controls from patients with no history of BC, and performing miRNA profiling of miRNAs from three different biosources, we have conducted a comprehensive attempt to investigate the biomarker potential of EVmiRNAs in BC patients. Additionally, we have conducted a replica EV/RNA isolation and sequencing run to strengthen the reliability of our results.

To explore the potential of miR-486-5p and miR-451a as biomarkers of recurrence-free survival, future studies should include samples from T1 patients with recurrence in order to detect a possible correlation between the expression of these two miRNAs and tumor recurrence. The VESCAN biobank at our local hospital (Biobank1, St. Olav’s University Hospital, Trondheim, Norway), is continuously collecting urine and serum samples for EV-analysis, enabling such a study in the future.

## 4. Materials and Methods

### 4.1. Clinical Samples

Urine and serum supernatant samples from 41 BC patients and 15 NCPs were provided by the regional research biobank. Samples were selected from patients recruited within the VESCAN project, which encompasses the collection of urine and serum samples before cystoscopy examinations and BC surgery, as well as at subsequent postsurgery check-ups. For the present study, we chose NMBC patients with the following characteristics: (1) Urine and serum samples were biobanked prior to BC surgery. (2) Urine and serum samples were biobanked prior to at least one postsurgery check-up where no recurrence was detected. (3) Patients had no other concurrent malignancies.

If patient samples were biobanked at multiple check-ups where no evidence of recurrence was detected, we included samples at two time points, an early (usually 3 months postsurgery) and late (usually 12 months postsurgery) check-up (Table S3). The NCPs had no history of BC or other malignancies and had donated urine and serum samples prior to a cystoscopy examination, where no evidence of malignancy was found. The use of all patient material in this study was approved by the Regional Ethical Committee (REK 2017/2367).

### 4.2. Biobanking Procedure

Urine samples were centrifuged at 500× *g* for 15 min, the supernatant of which was centrifuged again at 12,000× *g* for 30 min. Serum was centrifuged at 12,000× *g* for 30 min. After the 12,000× *g* step, urine and serum supernatants were stored at −80° at Biobank1 for subsequent EV isolation. Samples were collected during patients’ attendance at the Department of Urology, St. Olav’s University Hospital, Trondheim, Norway during April 2017 and March 2020.

### 4.3. Sequencing Study Design

The study design was comprised of two separate EV/RNA isolation and sequencing sets. Sequencing set 1 included 28 BC patients (18 stage Ta and 10 stage T1) and 15 NCPs. EV-contained total RNA was sequenced for urine and serum for all patients, while serum supernatant total RNA was sequenced for BC patients with stage T1 disease and NCPs. Sequencing set 2 consisted of 13 additional patients (9 stage Ta and 4 stage T1), as well as replicates for all T1 patient samples from sequencing set 1. Serum EVs were excluded from sequencing set 2, as results from sequencing set 1 revealed no significant findings in serum EVs for any of the patient groups.

### 4.4. Isolation of EVs and Small Non-Coding RNAs

EVs and EV-contained total RNA were isolated from biobanked urine and serum supernatants using the exoRNeasy kit (Qiagen, Düsseldorf, Germany) according to manufacturers’ instructions. Total RNA from serum supernatant was isolated with the miRNeasy kit (Qiagen). For the purpose of EV quantification, EVs were isolated from separate aliquots of urine and serum samples corresponding to the first sequencing set using the miRCURY Exosome Cell/Urine/CSF and miRCURY Exosome Serum/Plasma kits (Qiagen). Five milliliters of urine and 0.5 mL of serum supernatant were used for isolation for both kits. Prior to isolation, samples were centrifuged for 5 min at 3000× *g* in order to remove cryoprecipitates.

### 4.5. Nanoparticle Tracking Analysis

EVs were visualized and quantified through nanoparticle tracking analysis (NTA) on a Nanosight NS300 with 70 mW laser with wavelength 405 nm, Nanosight Ltd., Amesbury, UK. Urine samples were diluted in PBS at a ratio of 1:100, while serum samples were diluted 1:1000. Duplicate measurements were recorded for each sample. Video recording time for each measurement was chosen to be 60 s. NTA measurements were carried out on sequencing set 1 samples.

### 4.6. Small RNA Sequencing

sRNA sequencing libraries were prepared using the NEXTflex small RNA-seq kit v3 (Bioo Scientific, Austin, TX, USA). The adapter-dimer reduction technology incorporated into this kit allows low input library preparation. Reducing ligation-associated bias involves the use of adapters with randomized bases at the ligation junctions, resulting in a greatly decreased bias in comparison to standard protocols. In brief, the total RNA extracted from EVs from either urine and serum supernatant were used as a template for 3′ 4N and 5′ 4N adenylated adapter ligation, followed by reverse transcription-first strand synthesis. By applying these products as a template for second-strand synthesis, double-stranded cDNA was prepared by PCR amplification (20 cycles). Fragments/libraries were run on a Labchip GX (Caliper/PerkinElmer), for quality control and quantitation. Individual libraries were normalized to 10 nM and pooled. The library pool was purified with the QIAquick PCR Purification Kit (Qiagen AB, Sweden) according to instructions. Automated size selection was performed using the Blue Pippin (Sage Science, Beverly, MA, USA), with a range of135-170 bp to select the ~152 bp fragment. Following size selection, the pool was evaluated on Bioanalyzer (Agilent Technologies, Santa Clara, CA, USA) using the High Sensitivity DNA kit. The pool of libraries was quantified with the KAPA Library Quantification Kit (Roche, Pleasanton, CA, USA).

Quantitated libraries were further diluted and normalized to 2.4 nM (for Illumina HiSeq4000 Sequencing System) and 2.6 pM (for Illumina NextSeq Sequencing System), before clustering. Single read sequencing was performed for 51 cycles on three NextSeq500 HO flowcells and one HiSeq4000 flowcell, according to the manufacturer’s instructions (Illumina, Inc., San Diego, CA, USA). Sequence reads were demultiplexed and converted from BCL to FASTQ file format using bcl2fastq2 conversion software V2.20.0422 (Illumina, Inc. San Diego, CA, USA).

### 4.7. Statistical Analysis

FASTQ files were processed through the Unitas software (v1.7.5) [60] providing counts for the functionally different classes of small non-coding RNAs. Reference annotations were taken from Ensembl release 96 and miRBase release 22. The counts for miRNA were imported to the R package DESeq2 [61] and a variance stabilizing transformation was applied [62]. The 500 miRNAs with the highest variation were used to estimate the Principal Component Analysis (PCA). A linear mixed model was estimated to identify the differentially expressed genes between presurgery and postsurgery samples. The model was partitioned to account for variances with patients, biosource, and batch origins. The function getPredictedTargets with the “geom” method from the R package miRNAtap was used to predict gene targets for miRNAs and the topGO package was used to run GO term analysis Figures were generated using the ggplot2 R package.

## 5. Conclusions

In this study, urinary EVs, serum EVs and serum supernatant were investigated as potential reservoirs of BC-specific miRNA biomarkers. Only in urinary EVs did we find DEmiRNAs that could be verified when a replica EV/RNA isolation and sequencing run were taken into account. For BC patients with T1 disease, EV-contained miR-451a and miR-486-5p were found to be significantly upregulated in urine presurgery samples compared to postsurgery check-ups. Gene ontology analysis revealed that these miRNAs are involved in processes important in malignancies, such as cell differentiation and cell cycle regulation. MiR-451a and miR-486-5p may be potential biomarkers for recurrence-free survival in BC patients with stage T1 disease. Future studies that investigate the expression of these miRNAs in T1 patients with disease recurrence may elucidate their potential as BC biomarkers.

## Figures and Tables

**Table 1:** BC patient characteristics. Age at inclusion, sex, smoking status, primary BC diagnosis, and diagnosis at the time of inclusion into the study (TOI) for BC patients. Diagnosis is denoted by stage (Ta and T1), with Ta tumors being confined to the epithelial lining of the bladder, while T1 tumors have invaded submucosal tissue. The degree of cellular differentiation within the tumor is denoted by grade (G1-G3), with higher grades representing less differentiated tumors. If patients had been diagnosed with BC prior to inclusion in the study, the year of diagnosis is shown. If patients received their primary diagnosis upon inclusion in the study, it is found in the diagnosis at the time of inclusion column.

	Sex	Age	Smoking Status	Primary Diagnosis	Diagnosis at TOI
Patient 1	M	74	PREVIOUS	TaG1	TaG1
Patient 2	M	76	PREVIOUS	TaG1	TaG1
Patient 3	F	77	PREVIOUS	TaG1 (2015)	TaG1
Patient 4	M	61	PREVIOUS	TaG1	TaG1
Patient 5	M	62	NEVER	TaG1	TaG1
Patient 6	M	59	PREVIOUS	TaG1	TaG1
Patient 7	F	61	PREVIOUS	TaG2	TaG2
Patient 8	M	52	PREVIOUS	TaG2	TaG2
Patient 9	M	81	NEVER	TaG2 (2016)	TaG2
Patient 10	M	81	PREVIOUS	TaG2 (2012)	TaG2
Patient 11	M	82	PREVIOUS	TaG2	TaG2
Patient 12	M	72	PREVIOUS	TaG1	TaG1
Patient 13	M	72	PREVIOUS	TaG1 (1990)	TaG1
Patient 14	M	65	PREVIOUS	TaG1	TaG1
Patient 15	M	65	PREVIOUS	TaG2 (2016)	TaG2
Patient 16	M	72	PREVIOUS	TaG2 (2011)	T1G3
Patient 17	M	72	PREVIOUS	T1G3	T1G3
Patient 18	M	74	NEVER	T1G2	T1G2
Patient 19	M	70	NEVER	T1G3	T1G3
Patient 20	M	82	NEVER	T1G3	T1G3
Patient 21	M	47	NEVER	T1G3/Carcinoma in-situ	T1G3/Carcinoma in-situ
Patient 22	M	72	PREVIOUS	T1G3	T1G3
Patient 23	F	70	NEVER	T1G3	T1G3
Patient 24	F	70	NEVER	T1G2	T1G2
Patient 25	M	81	PREVIOUS	TaG2 (2005)	TaG2
Patient 26	M	81	PREVIOUS	TaG3	TaG3
Patient 27	M	75	PREVIOUS	T1G2	T1G2
Patient 28	F	80	PREVIOUS	TaG2 (2012)	TaG2
Patient 29	F	65	PREVIOUS	TaG1	TaG1
Patient 30	M	82	NEVER	TaG2	TaG2
Patient 31	F	83	NEVER	TaG1	TaG1
Patient 32	F	71	NEVER	TaG2	TaG2
Patient 33	F	77	PREVIOUS	TaG2 (2008)	TaG2
Patient 34	M	75	NEVER	TaG2	TaG2
Patient 35	M	79	CURRENT	TaG2	TaG2
Patient 36	M	79	PREVIOUS	TaG1	TaG1
Patient 37	M	69	PREVIOUS	TaG2	TaG2
Patient 38	M	70	PREVIOUS	TaG3 (2018)	T1G3
Patient 39	M	64	PREVIOUS	T1G3	T1G3
Patient 40	M	78	PREVIOUS	T1G3	T1G3
Patient 41	M	89	PREVIOUS	T1G2	T1G2

**Table 2:** Number of patients for each DEmiRNA identified in urine EVs and serum supernatants. Tables A and B show the number out of the fourteen T1 patients where the expression was higher in the presurgery vs. postsurgery for the 11 DEmiRNA from urine EVs (Figure 2D) and for the 12 DEmiRNA from serum supernatant (Figure 2F).

(A) Urine EVs		(B) Serum Supernatant	
miR-486-5p	12	miR-3158-3p	9
miR-451a	12	miR-3921-3p	7
miR-205-5p	11	miR-4654-3p	9
miR-1246	10	miR-922-3p	11
miR-483-3p	12	miR-612-3p	7
miR-1323	14	miR-4453-3p	8
miR-6073-3p	11	miR-942-3p	7
miR-202-3p	8	miR-4734-3p	9
miR-4508-5p	8	miR-185-3p	8
miR-483-5p	9	miR-6851-5p	9
miR-6768-5p	8	miR-135a-1-3p	3
		miR-3153-3p	3

## Data Availability

The data presented in this study are available upon request from the corresponding author.

## Supplementary Materials

Supplementary materials can be found at https://www.mdpi.com/article/10.3390/cimb43010024/s1.
