# Supplementary material for: Differentially Expressed Extracellular Vesicle-Contained microRNAs before and after Transurethral Resection of Bladder Tumors
Source: Curr Issues Mol Biol. 2021 Jun 4;43(1):286–300. doi: 10.3390/cimb43010024 (PMC8929081; doi:10.3390/cimb43010024)
Supplement: Supplementary file 1 [file cimb-43-00024-s001.zip › cimb-1206810-supplementary.pdf]

Supplementary

	Sex	Age	Smoking status	Initial Diagnosis	Urine EVs particle concentration	Serum EVs particle concentration	urine Evs	Serum Evs
**NCP 1**	M	72	CURRENT	Adenometous hyperplasia	2.95e+007 +/- 2.56e+006 particles/ml	7.20e+008 +/- 6.47e+007 particles/ml	79.5	80.8
**NCP 2**	M	70	PREVIOUS	Badder diverticulum with chronic inflammation	1.48e+008 +/- 1.41e+007 particles/ml	2.26e+008 +/- 2.85e+007 particles/ml	87.1	82
**NCP 3**	F	44	NEVER	Normal cystoscopy	4.97e+007 +/- 1.17e+006 particles/ml	2.57e+008 +/- 5.66e+005 particles/ml	86.4	76.9
**NCP 4**	M	67	NEVER	Chronic inflammation	1.35e+008 +/- 1.22e+006 particles/ml	1.53e+008 +/- 4.12e+007 particles/ml	63.7	69.7
**NCP 5**	F	52	NEVER	Inverted urothelial papilloma	No result	1.80e+008 +/- 1.22e+007 particles/ml	-	68
**NCP 6**	F	52	CURRENT	Chronic inflammation	1.33e+008 +/- 5.74e+006 particles/ml	1.59e+008 +/- 4.26e+007 particles/ml	97	-
**NCP 7**	F	73	NEVER	Glanudlar cystic cystitis	4.60e+008 +/- 9.82e+007 particles/ml	2.14e+008 +/- 1.05e+007 particles/ml	96.8	78.3
**NCP 8**	F	70	NEVER	Chronic inflammation	2.34e+008 +/- 4.14e+007 particles/ml	2.49e+008 +/- 8.89e+006 particles/ml	89.1	-
**NCP 9**	M	82	PREVIOUS	Papilloma with local inflammation	1.61e+008 +/- 6.49e+005 particles/ml	1.95e+008 +/- 5.19e+007 particles/ml	108.8	64.8
**NCP 10**	F	79	PREVIOUS	Reactive squamous epithial metaplasia	5.57e+008 +/- 2.06e+007 particles/ml	1.89e+008 +/- 3.19e+005 particles/ml	101.8	93.3
**NCP 11**	F	59	PREVIOUS	Papilloma	2.82e+008 +/- 9.83e+006 particles/ml	3.65e+008 +/- 3.07e+006 particles/ml	110	77.2
**NCP 12**	F	42	PREVIOUS	Chronic inflammation	1.13e+008 +/- 5.23e+007 particles/ml	2.95e+008 +/- 5.34e+006 particles/ml	134.5	126
**NCP 13**	M	46	CURRENT	Maloplakia	7.86e+008 +/- 1.33e+008 particles/ml	4.81e+008 +/- 1.73e+007 particles/ml	108.6	91.7
**NCP 14**	M	72	PREVIOUS	Glandular cystic cystitis	6.25e+008 +/- 7.87e+007 particles/ml	3.24e+008 +/- 2.31e+007 particles/ml	89.3	76.7
**NCP 15**	M	72	PREVIOUS	Chronic inflammation	2.07e+008 +/- 2.12e+007 particles/ml	2.62e+008 +/- 5.53e+007 particles/ml	69.9	126.6

Table S1. Characterization of NCPs and EV particle concentrations for urine and serum samples included in the study. Sex, age and smoking status for each NCP as well as their primary diagnosis upon inclusion into the study are outlined. Individual urine EV and serum EV particle concentration (particles/ml) and mode size (nm) by NTA are shown**.** Some size values are absent due to sample limitation.

	Sample Number	BCG Treatment
**Patient 15**	Sample 1	None
	Sample 2	Induction treatment
	Sample 3	None
	Sample 4	Maintenance treatment
**Patient 19**	Sample 1	None
	Sample 2	Induction treatment
	Sample 3	Maintenance treatment
**Patient 22**	Sample 1	None
	Sample 2	Induction treatment
	Sample 3	Maintenance treatment
**Patient 24**	Sample 1	None
	Sample 2	Induction treatment
**Patient 25**	Sample 1	None
	Sample 2	Induction treatment
**Patient 26**	Sample 1	None
	Sample 2	Induction treatment
**Patient**	Sample 1	None
	Sample 2	Induction treatment
**Patient**	Sample 1	None
	Sample 2	Induction treatment

Table S2. BCG treatment of BC patients. Table of patients who received BCG treatment during the study. When type of BCG treatment is noted next to sample number, this indicates treatment in the timeframe prior to the sample collection. Breakdown of specific patients and their detailed samples who underwent BCG treatment.

	Sample Number	Procedure	Clinical status	Clinical timepoint	Urine EVs particle concentration	Serum EVs particle concentration	Urine Evs particle size	Serum Evs particle size
**Patient 1**	Sample 1	TURB	TaG1	0	2.34e+008 +/- 1.09e+007	9.17e+008 +/- 1.28e+006	123.4	84.4
	Sample 2	Cystoscopy	Recurrence-free	6	8.67e+008 +/- 4.04e+007	3.63e+008 +/- 1.16e+007	96.3	65.8
	Sample 3	Cystoscopy	Recurrence-free	12	1.35e+008 +/- 7.81e+006	2.95e+008 +/- 3.92e+007	110.3	69.5
**Patient 2**	Sample 1	TURB	TaG1	0	3.64e+007 +/- 5.49e+006	2.25e+008 +/- 3.95e+007	117.2	78.7
	Sample 2	Cystoscopy	Recurrence-free	8	4.13e+008 +/- 2.21e+007	6.45e+007 +/- 1.39e+007	101	93.3
	Sample 3	Cystoscopy	Recurrence-free	21	6.99e+007 +/- 2.90e+006	1.52e+008 +/- 3.30e+007	111	96.7
**Patient 3**	Sample 1	TURB	TaG1	0	1.53e+008 +/- 1.50e+007	1.81e+008 +/- 5.04e+006	86.2	86.6
	Sample 2	Cystoscopy	Recurrence-free	3	7.15e+007 +/- 3.23e+006	6.24e+007 +/- 1.38e+007	93.9	131.9
	Sample 3	Cystoscopy	Recurrence-free	12	1.14e+008 +/- 6.01e+006	8.85e+007 +/- 6.21e+006	98.6	27.4
**Patient 4**	Sample 1	TURB	TaG1	0	1.60e+008 +/- 1.86e+007	3.04e+007 +/- 4.80e+006	70.5	57
	Sample 2	Cystoscopy	Recurrence-free	3	5.41e+008 +/- 1.57e+007	4.68e+007 +/- 3.32e+006	87	72.6
	Sample 3	Cystoscopy	Recurrence-free	12	7.04e+008 +/- 4.92e+007	1.62e+008 +/- 3.90e+006	86.6	95.6
**Patient 5**	Sample 1	TURB	TaG1	0	5.13e+008 +/- 2.83e+006	2.24e+008 +/- 1.30e+007	90.7	75.5
	Sample 2	Cystoscopy	Recurrence-free	3	2.42e+008 +/- 1.15e+008	3.59e+007 +/- 9.95e+006	75.4	70.3
	Sample 3	Cystoscopy	Recurrence-free	12	3.12e+008 +/- 7.61e+006	1.37e+008 +/- 3.55e+006	106	82.8
**Patient 6**	Sample 1	TURB	TaG1	0	6.60e+007 +/- 3.06e+006	3.79e+008 +/- 1.65e+008	88.3	67
	Sample 2	Cystoscopy	Recurrence-free	3	6.13e+007 +/- 1.49e+007	4.24e+008 +/- 2.73e+007	84.1	64.1
	Sample 3	Cystoscopy	Recurrence-free	12	1.29e+008 +/- 3.52e+007	5.93e+008 +/- 9.59e+007	93.5	70.3
**Patient 7**	Sample 1	TURB	TaG2	0	2.47e+008 +/- 4.65e+007	4.53e+008 +/- 5.08e+007	83.4	87.6
	Sample 2	Cystoscopy	Recurrence-free	6	3.28e+008 +/- 2.40e+007	1.10e+009 +/- 1.35e+008	82.8	82.4
	Sample 3	Cystoscopy	Recurrence-free	12	1.10e+008 +/- 4.79e+006	8.09e+008 +/- 2.44e+008	81.8	83.6
**Patient 8**	Sample 1	TURB	TaG2	0	2.17e+007 +/- 4.41e+006	9.24e+008 +/- 3.86e+007	134.6	69.3
	Sample 2	Cystoscopy	Recurrence-free	3	4.17e+007 +/- 8.65e+006	9.98e+008 +/- 5.85e+007	113.8	72.8
	Sample 3	Cystoscopy	Recurrence-free	12	3.09e+007 +/- 1.65e+006	7.65e+008 +/- 4.90e+007	68.3	82.1
**Patient 9**	Sample 1	TURB	TaG2	0	9.07e+007 +/- 6.98e+005	7.48e+008 +/- 2.00e+007	96.7	68.6

	Sample 2	Cystoscopy	Recurrence-free	3	3.62e+007 +/- 5.86e+006	5.52e+008 +/- 7.27e+007	68.6	71.4
	Sample 3	Cystoscopy	Recurrence-free	12	1.27e+008 +/- 6.14e+006	4.49e+008 +/- 8.48e+007	90.7	71.4
**Patient 10**	Sample 1	TURB	TaG2	0	4.85e+007 +/- 4.17e+006	7.01e+008 +/- 5.82e+007	28,6	66.7
	Sample 2	Cystoscopy	Recurrence-free	3	6.89e+007 +/- 4.79e+006	8.70e+008 +/- 5.76e+007	25,4	63.1
	Sample 3	Cystoscopy	Recurrence-free	6	3.80e+008 +/- 1.71e+007	2.44e+008 +/- 1.70e+007	105.8	70.4
**Patient 11**	Sample 1	TURB	TaG2	0	9.06e+007 +/- 1.56e+006	2.97e+008 +/- 1.50e+007	103.4	60.4
	Sample 2	Cystoscopy	Recurrence-free	7	2.12e+008 +/- 8.53e+005	5.45e+008 +/- 2.45e+007	97.4	64.3
	Sample 3	Cystoscopy	Recurrence-free	11	7.31e+008 +/- 4.59e+007	3.27e+008 +/- 7.77e+007	105	78.4
**Patient 12**	Sample 1	TURB	TaG1	0	5.94e+007 +/- 7.00e+005	4.48e+008 +/- 4.14e+006	102.9	66.4
	Sample 2	Cystoscopy	Reccurrence-free	7	2.73e+008 +/- 3.38e+007	3.90e+008 +/- 1.19e+008	95.2	67.5
	Sample 3	Cystoscopy	Recurrence-free	10	1.70e+008 +/- 7.05e+006	5.21e+008 +/- 2.71e+007	78.3	72.3
**Patient 13**	Sample 1	TURB	TaG1	0	4.46e+008 +/- 5.44e+007	3.59e+008 +/- 4.55e+007	129.6	53.9
	Sample 2	Cystoscopy	Recurrence-free	3	4.11e+008 +/- 3.90e+007	1.64e+008 +/- 5.37e+007	91.2	79.5
**Patient 14**	Sample 1	TURB	TaG1	0	9.70e+007 +/- 9.86e+006	6.48e+008 +/- 4.24e+007	142	60.9
	Sample 2	Cystoscopy	Recurrence-free	3	6.22e+007 +/- 3.87e+006	6.48e+008 +/- 4.24e+007	77.4	-
	Sample 3	Cystoscopy	Recurrence-free	7	1.60e+008 +/- 1.19e+007	3.73e+008 +/- 5.42e+007	88.7	86.2
**Patient 15**	Sample 1	TURB	TaG2	0	2.02e+008 +/- 1.71e+007	2.58e+008 +/- 8.58e+006	94.8	69.2
	Sample 2	Cystoscopy	Recurrence-free	6	3.56e+008 +/- 1.17e+006	2.21e+008 +/- 7.26e+006	117.2	67
	Sample 3	Cystoscopy	Recurrence-free	17	3.21e+009 +/- 7.99e+006	3.47e+008 +/- 1.00e+007	122.3	67.2
**Patient 16**	Sample 1	TURB	T1G3	0	1.69e+008 +/- 3.35e+007	3.11e+008 +/- 1.21e+007	112.1	65.6
	Sample 2	Cystoscopy	Recurrence-free	6	2.87e+007 +/- 2.22e+006	3.69e+008 +/- 3.93e+007	123.3	66
**Patient 17**	Sample 1	TURB	T1G3	0	2.82e+008 +/- 8.16e+007	6.84e+008 +/- 8.52e+006	86.1	62.7
	Sample 2	Clinical check-up + urography	Recurrence-free	5	1.57e+009 +/- 7.59e+006	8.92e+008 +/- 7.66e+007	106.3	68.6
**Patient 18**	Sample 1	TURB	T1G2	0	1.44e+008 +/- 3.19e+006	7.26e+008 +/- 2.82e+007	138.8	63.7
	Sample 2	Cystoscopy	Recurrence-free	5	7.44e+008 +/- 2.36e+00	5.01e+008 +/- 1.99e+008	-	69
**Patient 19**	Sample 1	TURB	T1G3	0	3.51e+008 +/- 4.89e+007	7.08e+008 +/- 2.60e+007	104.1	73.5
	Sample 2	Cystoscopy	Recurrence-free	5	9.64e+007 +/- 9.13e+006	8.62e+008 +/- 1.24e+007	66.8	72.2
	Sample 3	Cystoscopy	Recurrence-free	13	4.18e+007 +/- 7.01e+006	4.49e+008 +/- 4.18e+007	85.5	61.7

**Patient 20**	Sample 1	TURB	T1G3	0	6.51e+008 +/- 1.72e+008	4.18e+008 +/- 3.22e+007	83.7	60
	Sample 2	Cystoscopy	Recurrence-free	5	2.36e+008 +/- 4.73e+007	1.01e+008 +/- 3.66e+007	105	77.4
	Sample 3	Cystoscopy	Recurrence-free	12	7.02e+007 +/- 1.82e+006	2.05e+008 +/- 1.97e+007	85.6	69.4
**Patient 21**	Sample 1	TURB	T1G3/CIS	0	8.16e+007 +/- 1.87e+007	6.54e+008 +/- 5.33e+007	64.5	64.3
	Sample 2	CT (chest, abdominal & pelvic)	Recurrence-free	6	6.20e+008 +/- 5.83e+00	1.31e+008 +/- 1.09e+007	-	91.1
**Patient 22**	Sample 1	TURB	T1G3	0	4.94e+007 +/- 5.72e+006	2.14e+008 +/- 2.13e+006	123.7	85.5
	Sample 2	Cystoscopy	Recurrence-free	4	7.91e+007 +/- 1.33e+007	4.81e+007 +/- 5.58e+006	101.1	-
	Sample 3	Cystoscopy	Recurrence-free	16	1.07e+008 +/- 2.71e+006	9.91e+007 +/- 9.68e+006	72	114.1
**Patient 23**	Sample 1	TURB	T1G3	0	2.58e+008 +/- 2.64e+006	4.81e+008 +/- 1.73e+007	101.9	76.9
	Sample 2	Cystoscopy	Recurrence-free	3	5.09e+008 +/- 1.08e+008	8.66e+007 +/- 4.49e+006	83.6	91.7
**Patient 24**	Sample 1	TURB	T1G2	0	3.46e+008 +/- 6.39e+007	1.89e+008 +/- 3.19e+005	99.9	75.9
	Sample 2	Cystoscopy	Recurrence-free	3	3.36e+008 +/- 1.10e+007	2.10e+008 +/- 2.41e+007	93.3	75.3
**Patient 25**	Sample 1	TURB	TaG2	0	2.14e+008 +/- 2.73e+007	1.30e+008 +/- 1.70e+007	119.1	77.2
	Sample 2	Cystoscopy	Recurrence-free	3	2.37e+008 +/- 9.05e+006	1.11e+008 +/- 1.46e+007	111.8	-
**Patient 26**	Sample 1	TURB	TaG3	0	6.39e+007 +/- 1.59e+007	2.49e+008 +/- 8.89e+006	95.4	89.2
	Sample 2	Cystoscopy	Recurrence-free	5	8.37e+008 +/- 2.10e+006	1.92e+008 +/- 9.22e+005	94.2	89.4
**Patient 27**	Sample 1	TURB	T1G2	0	3.31e+008 +/- 1.06e+007	2.95e+008 +/- 5.34e+006	101.7	71.6
	Sample 2	Cystoscopy	Recurrence-free	4	1.88e+008 +/- 3.40e+007	2.12e+008 +/- 1.99e+007	80.4	87
**Patient 28**	Sample 1	TURB	TaG2	0	1.13e+008 +/- 5.23e+007	1.59e+008 +/- 4.26e+007	95	97.2
	Sample 2	Cystoscopy	Recurrence-free	9	5.65e+008 +/- 1.31e+006	1.54e+008 +/- 1.49e+007	115	68
	Sample 3	Cystoscopy	Recurrence-free	12	1.01e+008 +/- 1.15e+007	1.11e+008 +/- 1.46e+007	105.4	77.9
Patient 29	Sample 1	TURB	TaG1	0	-	-	-	-
	Sample 2	Cystoscopy	Recurrence-free	3	-	-	-	-
Patient 30	Sample 1	TURB	TaG2	0	-	-	-	-
	Sample 2	Cystoscopy	Recurrence-free	3	-	-	-	-
Patient 31	Sample 1	TURB	TaG1	0	-	-	-	-
	Sample 2	Cystoscopy	Recurrence-free	3	-	-	-	-
Patient 32	Sample 1	TURB	TaG2	0	-	-	-	-
	Sample 2	Cystoscopy	Recurrence-free	3	-	-	-	-
Patient 33	Sample 1	TURB	TaG2	0	-	-	-	-
	Sample 2	Cystoscopy	Recurrence-free	3	-	-	-	-
	Sample 1	TURB	TaG2	0	-	-	-	-

Patient 34	Sample 2	Cystoscopy	Recurrence-free	3	-	-	-	-
Patient 35	Sample 1	TURB	TaG2	0	-	-	-	-
	Sample 2	Cystoscopy	Recurrence-free	3	-	-	-	-
Patient 36	Sample 1	TURB	TaG1	0	-	-	-	-
	Sample 2	Cystoscopy	Recurrence-free	3	-	-	-	-
Patient 37	Sample 1	TURB	TaG2	0	-	-	-	-
	Sample 2	Cystoscopy	Recurrence-free	3	-	-	-	-
Patient 38	Sample 1	TURB	T1G3	0	-	-	-	-
	Sample 2	Clinical check-up + urography	Recurrence-free	4	-	-	-	-
Patient 39	Sample 1	TURB	T1G3	0	-	-	-	-
	Sample 2	Cystoscopy	Recurrence-free	3	-	-	-	-
Patient 40	Sample 1	TURB	T1G3	0	-	-	-	-
	Sample 2	Cystoscopy	Recurrence-free	3	-	-	-	-
Patient 41	Sample 1	TURB	T1G2	0	-	-	-	-
	Sample 2	Cystoscopy	Recurrence-free	3	-	-	-	-

Table S3. Characterization of patient samples included in the study. Detailed breakdown for each of the 41 patients encompassing their presurgery and postsurgery samples included in the study. The table outlines the procedures undertaken for all samples as well as the clinical information and time point (months) for presurgery and postsurgery sample. Individual urine and serum EV particle concentration (particles/ml) and mode size (nm) by NTA are outlined. Some size values are absent due to sample limitation.

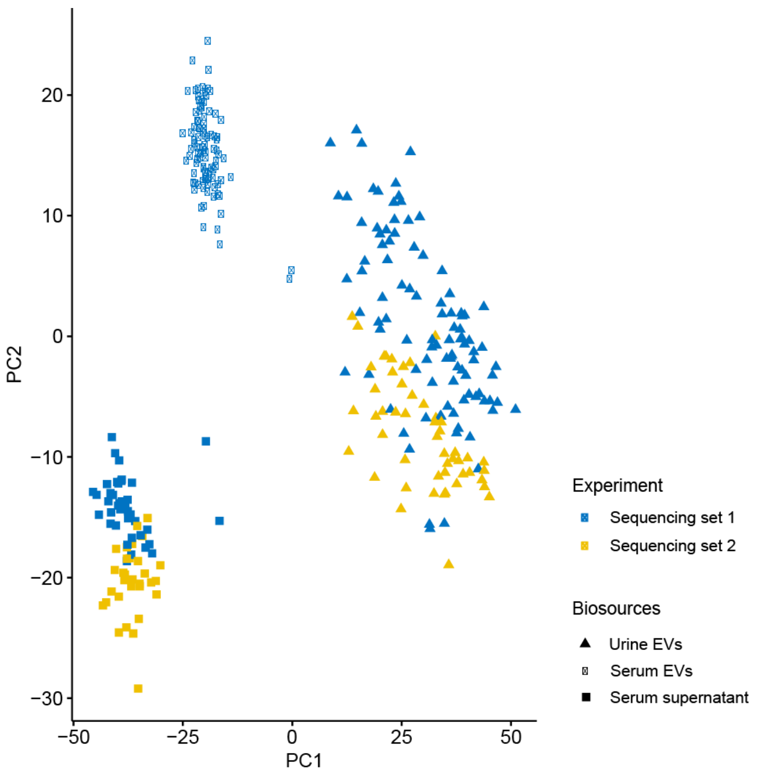

Figure S1. Principal component analysis our three biosources in sequencing set 1 & 2. The PCA plot depicts clear individual clustering for urine EVs, serum EVs and serum supernatant biosources representing all patient samples investigated in the both sequencing set 1 and sequencing set 2.

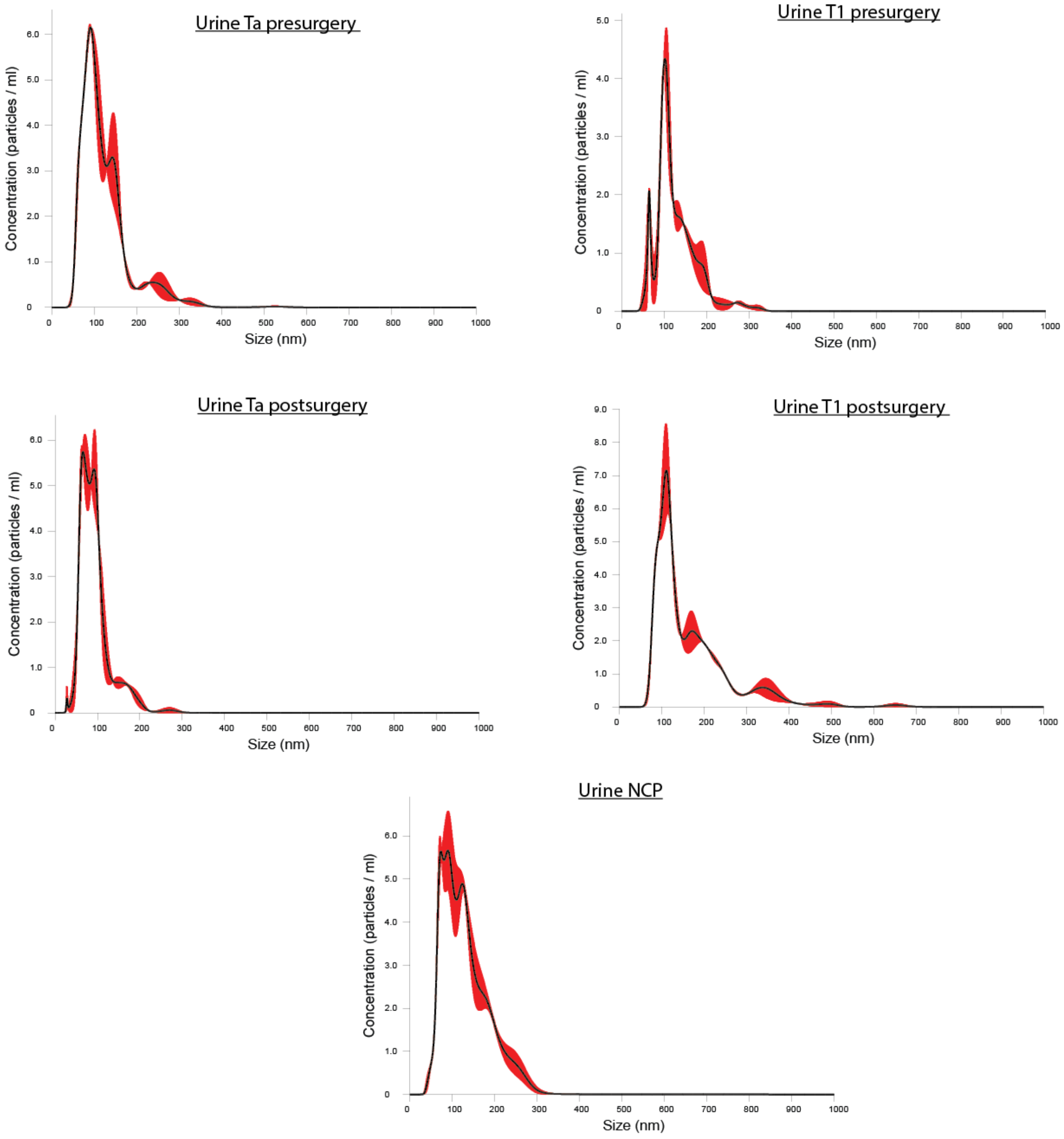

Figure S2. Representative NTA results for urine T1, Ta (presurgery and postsurgery) and NCP samples: NTA analysis graphs of EV samples isolated from urine depicting EV sizes representative of small EVs.

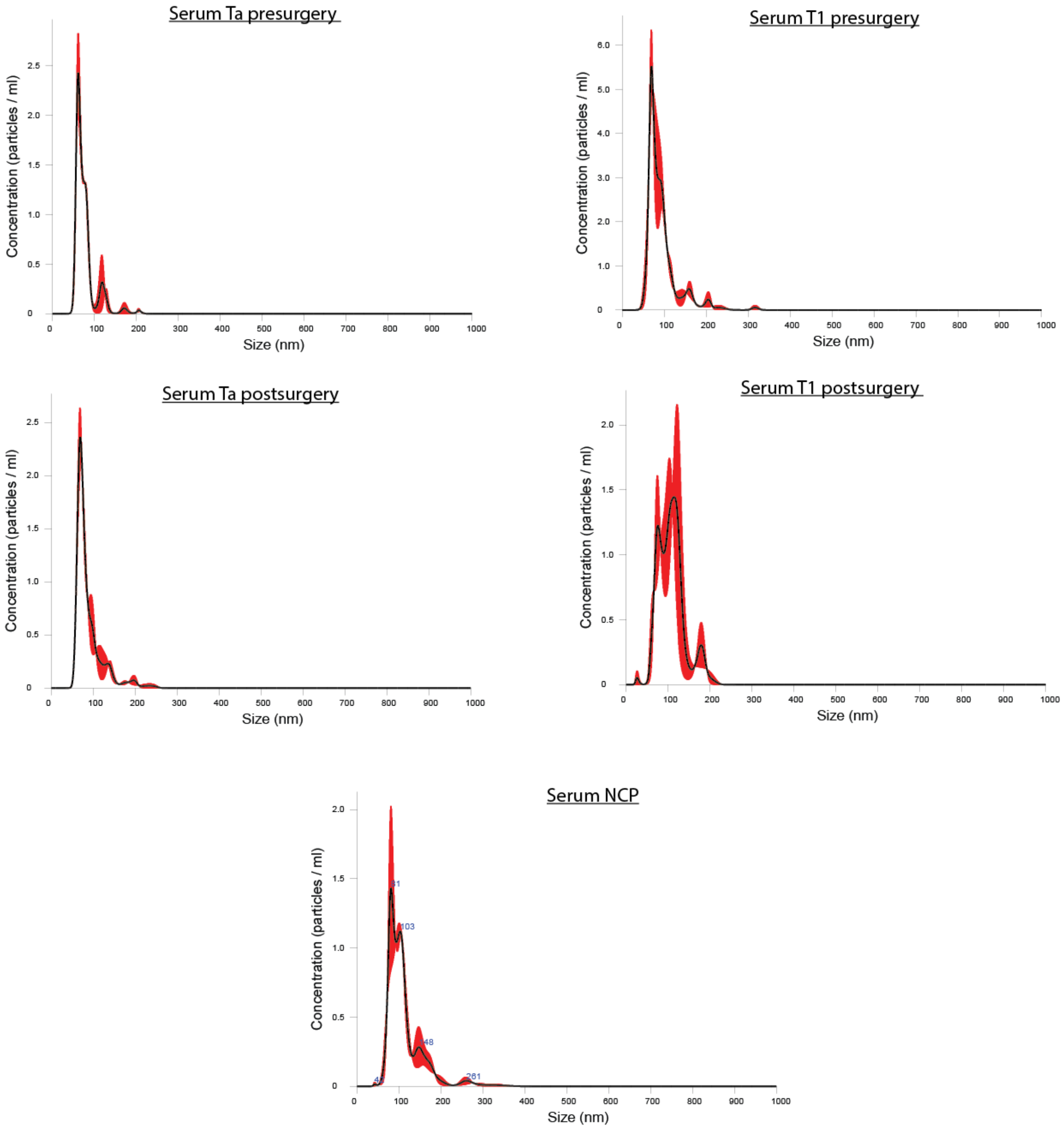

Figure S3. Representative NTA results for serum T1, Ta (presurgery and portsugery) and NCP samples: NTA analysis graphs of EV samples isolated from serum depicting EV sizes representative of small-EVs.

A. Urine EVs

T1 pre vs post

	Adj. p-value	Log2 fold change
miR-486-5p	1,90E-06	3,68E+00
miR-451a	3,86E-04	4,01E+00
miR-205-5p	3,75E-02	2,11E+00
miR-1246	3,75E-02	2,39E+00
miR-483-3p	3,60E-02	2,35E+00
miR-1323	3,60E-02	2,60E+00
miR-6073-3p	3,60E-02	2,24E+00
miR-202-30	3,75E-02	1,75E+00
miR-4508-5p	3,75E-02	1,64E+00
miR-483-5p	3,75E-02	2,02E+00
miR-6768-5p	3,66E-02	1,92E+00

B. Serum Supernatant

Adj. p-value

	T1 pre vs post	T1 pre vs NCP
miR-3158-3p	3,00E-07	1,20E-01
miR-3921-3p	3,47E-02	1,56E-02
miR-4654-3p	3,47E-02	1,20E-01
miR-922-3p	4,30E-02	1,20E-01
miR-612-3p	3,47E-02	1,70E-01
miR-4453-3p	7,97E-03	9,97E-01
miR-942-3p	7,97E-03	2,78E-01
miR-4734-3p	4,30E-02	5,50E-01
miR-185-3p	2,80E-02	5,60E-01
miR-6851-3p	3,47E-02	5,14E-01
miR-135a-1-3p	4,30E-02	9,76E-01
miR3153-3p	1,82E-01	1,71E-02

Log2 fold change

	T1 pre vs post	T1 pre vs NCP
miR-3158-3p	4,32E+00	2,72E+00
miR-3921-3p	2,76E+00	3,48E+00
miR-4654-3p	2,94E+00	2,87E+00
miR-922-3p	2,63E+00	2,47E+00
miR-612-3p	2,72E+00	2,30E+00
miR-4453-3p	2,52E+00	4,54E-03
miR-942-3p	3,25E+00	2,01E+00
miR-4734-3p	2,85E+00	1,27E+00
miR-185-3p	3,22E+00	1,24E+00
miR-6851-3p	3,15E+00	1,43E+00
miR-135a-1-3p	-2,65E+00	1,41E-01
miR3153-3p	-2,43E+00	-3,91E+00

Table S4. Adjusted p-values and log2 fold change for DEmiRNAs in urine EVs and serum supernatants. Adjusted p-values for all DEmiRNAs detected in (A) urine EVs T1 presurgery (pre) samples versus postsurgery (post) samples for the same patient and (B) serum supernatant T1 presurgery versus postsurgery or NCP samples as detected in Figure 2E and 2G.

Urine EVs : T1 pre vs post
Adj. P-value

	Seq set 1	Replica
miR-451a	3,86E-04	3,54E-05
miR-486-5p	1,92E-06	2,33E-02
miR-483-5p	3,75E-02	5,40E-02
miR-205-5p	3,75E-02	1,04E-01
miR-183-5p	1,38E-01	6,73E-02
miR-4508-5p	3,75E-02	2,16E-01
miR-483-3p	3,60E-02	5,01E-01
miR-126-3p	5,58E-01	2,69E-02
miR-16-5p	4,03E-01	2,06E-01
miR-320c-1-3p	1,38E-01	5,01E-01

Urine EVs : T1 pre vs post
Log2 fold change

	Seq set 1	Replica
miR-451a	4,01E+00	4,62E+00
miR-486-5p	3,68E+00	3,03E+00
miR-483-5p	2,02E+00	3,94E+00
miR-205-5p	2,11E+00	2,97E+00
miR-183-5p	9,35E-01	1,43E+00
miR-4508-5p	1,64E+00	1,65E+00
miR-483-3p	2,35E+00	1,34E+00
miR-126-3p	1,35E+00	1,96E+00
miR-16-5p	1,37E+00	1,27E+00
miR-320c-1-3p	1,40E+00	1,25E+00

Table S5. Adjusted p-values and log2 fold change for top 10 DEmiRNAs in T1 patients from both runs. Adjusted p-values for top 10 DEmiRNAs in urine EVs T1 presurgery (pre) versus postsurgery (post) samples in the ten T1 patients in sequencing (seq) set 1 and the ten T1 replica samples (replica) in sequencing set 2 as per Figure 3C.
